# Urinary Volatile Organic Compounds for the Detection of Prostate Cancer

**DOI:** 10.1371/journal.pone.0143283

**Published:** 2015-11-24

**Authors:** Tanzeela Khalid, Raphael Aggio, Paul White, Ben De Lacy Costello, Raj Persad, Huda Al-Kateb, Peter Jones, Chris S. Probert, Norman Ratcliffe

**Affiliations:** 1 Department of Surgery and Cancer, Imperial College London, London, United Kingdom; 2 Institute of Translational Medicine, University of Liverpool, Liverpool, United Kingdom; 3 Institute of Biosensor Technology, University of the West of England, Bristol, United Kingdom; 4 Bristol Urological Institute, North Bristol NHS Trust, Bristol, United Kingdom; The Chinese University of Hong Kong, HONG KONG

## Abstract

The aim of this work was to investigate volatile organic compounds (VOCs) emanating from urine samples to determine whether they can be used to classify samples into those from prostate cancer and non-cancer groups. Participants were men referred for a trans-rectal ultrasound-guided prostate biopsy because of an elevated prostate specific antigen (PSA) level or abnormal findings on digital rectal examination. Urine samples were collected from patients with prostate cancer (n = 59) and cancer-free controls (n = 43), on the day of their biopsy, prior to their procedure. VOCs from the headspace of basified urine samples were extracted using solid-phase micro-extraction and analysed by gas chromatography/mass spectrometry. Classifiers were developed using Random Forest (RF) and Linear Discriminant Analysis (LDA) classification techniques. PSA alone had an accuracy of 62–64% in these samples. A model based on 4 VOCs, 2,6-dimethyl-7-octen-2-ol, pentanal, 3-octanone, and 2-octanone, was marginally more accurate 63–65%. When combined, PSA level and these four VOCs had mean accuracies of 74% and 65%, using RF and LDA, respectively. With repeated double cross-validation, the mean accuracies fell to 71% and 65%, using RF and LDA, respectively. Results from VOC profiling of urine headspace are encouraging and suggest that there are other metabolomic avenues worth exploring which could help improve the stratification of men at risk of prostate cancer. This study also adds to our knowledge on the profile of compounds found in basified urine, from controls and cancer patients, which is useful information for future studies comparing the urine from patients with other disease states.

## Introduction

Prostate cancer is the second most prevalent malignancy affecting men worldwide, and yet there is no reliable screening tool. Over 40,000 cases are detected in the UK each year [1], usually by a combination of digital rectal examination (DRE), serum prostate specific antigen (PSA) measurements and followed by trans-rectal ultrasound-guided (TRUS) prostate biopsy. Prostate cancer is the second most common cause of death from malignancy in the UK [2].

Serum PSA, at a cut-off of 4 ng/ml, is part of an FDA-approved screening programme in the USA [3]. However, PSA testing is not used for screening elsewhere because of its low sensitivity, estimated to be 21% for detecting any prostate cancer and 51% for detecting high-grade cancers (Gleason ≥8) with PSA cut-off values of 4.0 ng/ml [4]. The false negative rate is up to 20% at that cut-off [5, 6]. In screening, only 25 to 30% of men with elevated PSA levels between 4–10 ng/ml have prostate cancer [7, 8].

Screening based on PSA levels has led to anxiety for many men, who do not have prostate cancer and the over-diagnosis of slow-growing cancers that were unlikely to pose a significant risk to patients. Two recent large screening trials found no significant decrease in mortality from prostate cancer following PSA-based screening tests [9, 10].

Other potential blood and urine based biomarkers for prostate cancer include: prostate cancer antigen 3 [PCA3] [11], intracellular PSA [12], PSA derivatives [13], early prostate cancer antigen 2 [14], annexin A3 [15], the fusion gene TMPRSS2:ERG [16, 17], human kallikrein 2 [18] and the Engrailed-2 protein (EN2) [19, 20]. However, none of these are used for screening [21, 22]. Sarcosine was proposed as a biomarker for metastatic prostate cancer [23], but this finding has not been replicated [24–26].

Several studies have reported that dogs can be trained to detect skin, bladder, lung, breast and ovarian cancer from breath, tissue and urine samples [27–30]. Cornu *et al* trained a Belgian Mallinois to identify prostate cancer from control urine samples with a sensitivity and specificity of 91% [31]. Elliker *et al* trained two dogs to identify prostate cancer from control urine samples, however, they were unable to discriminate cancers from controls when presented with new samples in a double-blind test [32]. Taverna *et al* collected samples from 362 prostate cancer patients and 540 controls: 2 dogs were tested, dog 1 performed best with a sensitivity of 100% and specificity of 98.7% and dog 2 achieved sensitivity of 98.6% and specificity of 97.6% [33].

Following the proof of concept studies in dogs, researchers have also tested electronic nose technology to discriminate the odour of urine from patients with prostate cancer and controls, achieving a sensitivity of 71 to 82% and specificity of 67 to 93% [34, 35].

The odour signature of urine is produced by substances known as volatile organic compounds (VOCs), which can be separated and identified by gas chromatography/mass spectroscopy (GC/MS). In the present study, we have compared the VOC profiles of urine headspace from 102 patients with urological symptoms, 59 of whom had prostate cancer and 43 who did not. To the best of our knowledge, no extensive work has been published on this to date.

## Materials and Methods

Ethical approval for the study was obtained from the Wiltshire Research Ethics Committee (REC reference number 08/H0104/63; protocol SU/2008/2901, version 3 approved 09/06/2009) with R&D approval from the University Hospitals Bristol NHS Foundation Trust from where participants were recruited over a 13-month period. Each participant reviewed an information sheet and gave written consent. All participants were men who were referred for a TRUS guided prostate biopsy because of an elevated PSA level or abnormal findings on DRE, secondary to other urological problems (see S1 Table).

Urine samples were collected in universal bottles prior to the patients TRUS prostate biopsy (10–12 cores) and samples were classified as prostate cancer or controls after pathological examination of the biopsy specimens. Urinalysis and specific gravity were performed on all samples under the SOP of the urology clinic. Prostate specific antigen levels were measured at the Bristol Royal Infirmary using Immulite 2000 PSA assay and Immulite 2000 analyser (Siemens Medical Solutions Diagnostics, New York, USA). The demographics for the patient groups studied are given in Table 1.

**Table 1:** Demographics for study participants with and without prostate cancer.

	N	Age range in years (median)	PSA range (ng/mL) (median)	No. of smokers (%)
Controls	43	41–81 (63)	0.8–30 (6.2)	7 (16)
Prostate cancer	59	50–88 (69)	3.4–647 (10.2)	10 (17)

The comorbidities and medication of patients are included in S1 Table along with the reason for patient referral for a prostate biopsy. Patients were excluded if they had a history of urothelial carcinoma or other known malignancies, a urinary tract infection, or a urinary catheter in situ. There were no exclusion criteria regarding ethnicity of the patient, the consumption of alcohol, tobacco, drugs, or food. Aliquots of fresh urine, 0.75 ml, were transferred to septum topped headspace vials (Sigma Aldrich, Dorset, UK) and were frozen at -20°C until analysis. There is no evidence that storage at -20°C has a negative influence on the presence of VOCs in headspace gases from urine samples [36, 37]. In addition, samples were collected, stored and analysed randomly. Therefore, any variation due to sample storage would have a similar effect on both cancer and control groups studied here.

Prior to urine headspace analysis each sample was defrosted by immersing the vial in a water bath at 60°C for 30 seconds. One single aliquot of urine sample per patient was used for VOC analysis. Thereafter, each sample was treated with an equal volume (0.75 ml) of sodium hydroxide (1M; Fisher Scientific, Leicestershire, UK). The addition of base, acid and salt are commonly used methods for improving the detection of VOCs from urine samples [36–39]. In general, these methods increase the concentration of VOCs in the headspace by increasing the ionic strength of the sample. In this study, exactly the same treatment method was applied to urine samples from patients with cancer and controls. Therefore, we expect the effect of sodium hydroxide to be similar in both groups. The mixture was equilibrated at 60°C in a water bath for 30 minutes prior to, and during, extraction of VOCs from the headspace with a solid-phase micro-extraction (SPME) fibre.

The SPME fibre was 85 μm thick and consisted of carboxen/ polydimethylsiloxane (Sigma Aldrich, Dorset, UK). The fibre was exposed to the headspace above the urine mixture for 20 minutes and following extraction the VOCs were analysed by GC/MS (Perkin Elmer Clarus 500 quadrupole, Beaconsfield, UK). The VOCs were thermally desorbed from the fibre at 220°C in the injection port of the GC/MS for 5 minutes. Injection was made in splitless mode and a split of 50 ml/min was turned on two minutes into the run.

Helium carrier gas of 99.996% purity (BOC, Guildford, UK) was passed through a helium purification system, Excelasorb^™^ (Supelco, Poole, UK) at 1 ml min^-1^. The GC column was a 60 metre long Zebron ZB-624 capillary column with an inner diameter of 0.25 mm and a film thickness of 1.4 μm, specifically designed for the separation of VOCs (Phenomenex, Macclesfield, UK). Its composition consisted of 94% dimethyl polysiloxane and 6% cyanopropyl-phenyl.

The GC/MS temperature program of the run was as follows: initial oven temperature was held at 40°C for 2 minutes then the temperature was ramped up at a rate of 5°C/min to 220°C, with a 4 minute hold at this temperature to give a total run time of 42 minutes. The mass spectrometer was run in electron impact (EI) ionization mode, scanning the mass ion range 10–300 at 0.05 scan /sec. A 4 minute solvent delay was used at the start of the run.

### Data processing

The GC-MS data was processed using a pipeline involving the Automated Mass Spectral Deconvolution and Identification System software (AMDIS, Version 2.71, 2012), the NIST mass spectral library (version 2.0, 2011) and the R (R core team, 2013) package Metab [40]. AMDIS and NIST were used to build a VOC library containing 197 metabolites present in the urine samples analysed in this study (S2 Table). A forward and reverse match of 800/1000 and above was used for assigning tentative compound identifications. Using this VOC library, AMDIS was then applied for deconvoluting GC-MS chromatograms and identifying metabolites. The report generated by AMDIS was further processed by Metab in order to confirm the identity of metabolites and recalculate their relative abundances based on the intensity of a specific ion mass fragment per metabolite. In order to develop robust parsimonious statistical models, those compounds found to be present in fewer than 20% of the patients in both groups (i.e. relatively rare compounds) or present in more than 90% of the patients in both groups (i.e. relatively common volatiles) were removed from the data set before statistical modelling.

### Statistical Analysis

The VOC profiles of every patient were converted to binary data based on the presence and absence of metabolites. Then, four different approaches (Table 2) were applied for feature selection prior to model building.

**Table 2:** Approaches and R packages applied for feature selection prior to statistical modelling.

Description	R package::function	Reference
•Wrapper approach built around random forest	Boruta::Boruta	[41]
•Linear discriminant analysis with stepwise feature selection	caret::stepLDA	[42]
•Backwards selection of predictors based on predictor importance ranking	caret::rfe	[42]
•Wrapper approach built around bagging tree	caret::treebagFuncs	[42]

The features, or VOCs, selected by at least one feature selection approach were used for developing classifiers using both Fisher’s Linear Discriminant Analysis [43] and Breiman’s Random Forest [42] decision tree. These classifiers were evaluated as two distinct approaches. This process was then repeated utilizing VOCs selected by at least one feature selection approach and the PSA levels of each patient to see how results compare to that of VOCs alone and the PSA test alone.

**Fig 1:**
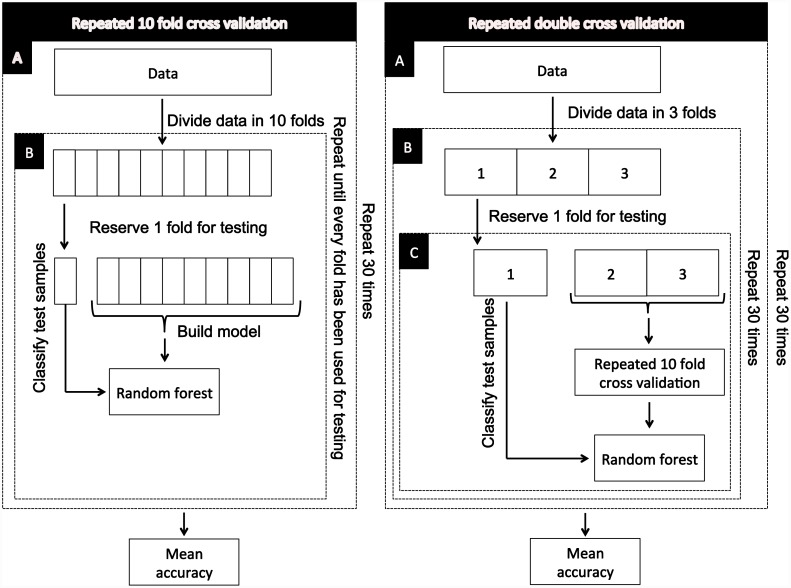
The pipeline of the validation techniques known as repeated 10 fold cross validation and repeated double cross validation. A Monte-Carlo variation of each technique is achieved by randomising the labels of the testing samples.

It is well-known that model building and model testing on the same data can produce biased results and can suffer from over fitting with models describing chance idiosyncratic sample features rather than real trends. This sample description approach is prone to inflated and optimistic measures of model accuracy, and much lower accuracy rates may be seen when the model is applied to fresh data [44, 45]. To overcome these potential flaws, model validation using repeated 10-fold cross-validation as described by Delen [46] was implemented. Repeated double cross-validation as described, implemented and strongly recommended by Filzmoser and colleagues [45], and by Anderssen and colleagues [44], was also used for model building and model assessment. Fig 1 gives a schematic overview of repeated 10-fold cross-validation and repeated double cross-validation. The cross-validation strategy was based on 30 repeats and ten-folds. The double cross-validation strategy also had 30 repeats of the outer loop (model evaluation loop), with calibration and test data based on 3 folds. The inner loop (the model tuning loop) used training and test data based on 10 folds and with 30 repeats.

It is instructive to demonstrate a class structure over any chance relationship. Based on the work of Ojala [47], the two cross-validation schemes were repeated on the data but with a Monte Carlo random permutation of class labels (prostate cancer, control) in each repeat. This permutation approach provides a reference level of chance agreement for the modelling process, which is needed to help quantify the effects captured by cross-validated models derived on authentic data. All modelling techniques and model validations were applied using the R package Caret [42].

## Results

The diagnostic capability of using PSA levels alone for this patient cohort was assessed. Using repeated 10-fold cross-validation, patients could be classified into prostate cancer and cancer-free groups based on their PSA levels with mean accuracies of 62% and 64%, using Random Forest and Linear Discriminant Analysis, respectively (Table 3).

**Table 3:** Accuracy results of PSA testing for prostate cancer assessed with repeated 10 fold cross validation of Random Forest and Linear Discriminant Analysis (LDA) models.

	Repeated 10 fold cross validation					
Model	Min.	1st.Qu.	Median	Mean	3rd.Qu.	Max.
**Random Forest**	0.10	0.50	0.60	0.62	0.70	1.00
**LDA**	0.27	0.56	0.64	0.64	0.70	1.00
	Monte-Carlo 10 fold cross validation					
Model	Min.	1st.Qu.	Median	Mean	3rd.Qu.	Max.
**Random Forest**	0.10	0.36	0.40	0.43	0.50	0.73
**LDA**	0.33	0.55	0.60	0.58	0.60	0.80

Using repeated double cross-validation, the mean accuracies fell to 61 and 63%, using Random Forest and Linear Discriminant Analysis, respectively (Table 4).

**Table 4:** Accuracy results of PSA testing for prostate cancer assessed using repeated double cross validation of Random Forest and Linear Discriminant Analysis (LDA) models.

	Repeated double cross validation											
	Accuracy				Sensitivity				Specificity			
Model	Mean	Min	Median	Max	Mean	Min	Median	Max	Mean	Min	Median	Max
**Random Forest**	0.61	0.43	0.61	0.74	0.66	0.35	0.65	0.90	0.53	0.21	0.57	0.71
**LDA**	0.63	0.53	0.63	0.74	0.87	0.58	0.89	1.00	0.31	0.07	0.29	0.71
	Monte-Carlo repeated double cross validation											
	Accuracy				Sensitivity				Specificity			
Model	Mean	Min	Median	Max	Mean	Min	Median	Max	Mean	Min	Median	Max
**Random Forest**	0.50	0.29	0.50	0.68	0.58	0.26	0.57	0.95	0.41	0.08	0.43	0.69
**LDA**	0.51	0.31	0.51	0.71	0.81	0.43	0.81	1.00	0.22	0.00	0.21	0.53

Based on the presence or absence of VOCs alone, urine samples could be classified with a mean accuracy of 66% with repeated 10-fold cross-validation (Table 5). Source data appear in S3 Table.

**Table 5:** Accuracy results of repeated 10 fold cross validation of the Random Forest and Linear Discriminant Analysis (LDA) models built to classify urine samples from patients with prostate cancer and cancer-free controls based on the presence or absence of VOCs.

	Repeated 10 fold cross validation					
Model	Min.	1st.Qu.	Median	Mean	3rd.Qu.	Max.
**Random Forest**	0.30	0.60	0.70	0.66	0.73	1.00
**LDA**	0.27	0.59	0.67	0.66	0.73	1.00
	Monte-Carlo 10 fold cross validation					
Model	Min.	1st.Qu.	Median	Mean	3rd.Qu.	Max.
**Random Forest**	0.00	0.45	0.50	0.51	0.60	0.73
**LDA**	0.10	0.44	0.50	0.50	0.60	0.70

Using repeated double cross-validation, the mean accuracies fell to 65 and 63%, using Random Forest and Linear Discriminant Analysis, respectively (Table 6).

**Table 6:** Accuracy results of repeated double cross validation of the Random Forest and Linear Discriminant Analysis (LDA) models built to classify urine samples from patients with prostate cancer and cancer-free controls based on the presence or absence of VOCs.

	Repeated double cross validation											
	Accuracy				Sensitivity				Specificity			
Model	Mean	Min	Median	Max	Mean	Min	Median	Max	Mean	Min	Median	Max
**Random Forest**	0.65	0.47	0.66	0.79	0.74	0.37	0.75	0.90	0.53	0.13	0.53	0.86
**LDA**	0.63	0.44	0.64	0.76	0.75	0.35	0.77	1.00	0.47	0.13	0.50	0.79
	Monte-Carlo repeated double cross validation											
	Accuracy				Sensitivity				Specificity			
Model	Mean	Min	Median	Max	Mean	Min	Median	Max	Mean	Min	Median	Max
**Random Forest**	0.50	0.30	0.51	0.64	0.63	0.25	0.64	0.93	0.37	0.07	0.37	0.72
**LDA**	0.50	0.26	0.50	0.67	0.65	0.25	0.67	0.92	0.35	0.05	0.33	0.76

Given that repeated double cross-validation is a much more rigorous technique of cross-validation than repeated 10-fold cross-validation, a reduction in test performance was expected. With a Monte Carlo random permutation of class labels, the mean accuracies fell to 50% using both modelling techniques. Therefore, the classification of samples based on the presence or absence of VOCs is somewhat better than what can be expected by chance alone. The final set of features selected and used to develop classifiers by both Fisher’s Linear Discriminant Analysis and Breiman’s Random Forest decision tree, included 2,6-dimethyl-7-octen-2-ol, pentanal, 3-octanone, and 2-octanone. Except for pentanal, all of these compounds were down-regulated and/or less frequently present in the urine samples from prostate cancer patients. The classification of patients into cancer and control groups based on these features in combination with PSA levels are summarised in Tables 7 and 8.

**Table 7:** Accuracy results of repeated 10 fold cross validation of the Random Forest and Linear Discriminant Analysis (LDA) models built to classify patients with prostate cancer and cancer-free controls based on blood PSA levels and urinary VOCs.

	Repeated 10 fold cross validation (%)					
Model	Min.	1st.Qu.	Median	Mean	3rd.Qu.	Max.
**Random Forest**	20.00	66.67	72.73	73.69	80.00	100.00
**LDA**	22.22	58.89	63.64	64.85	72.73	100.00
	Monte-Carlo 10 fold cross validation (%)					
Model	Min.	1st.Qu.	Median	Mean	3rd.Qu.	Max.
**Random Forest**	10.00	45.45	55.56	55.79	66.67	90.91
**LDA**	10.00	40.00	50.00	48.00	60.00	88.89

**Table 8:** Accuracy results of repeated double cross validation of the Random Forest and Linear Discriminant Analysis (LDA) models built to classify patients with prostate cancer and cancer-free controls based on blood PSA levels and urinary VOCs.

	Repeated double cross validation (%)											
	Accuracy				Sensitivity				Specificity			
Model	Mean	Min	Median	Max	Mean	Min	Median	Max	Mean	Min	Median	Max
**Random Forest**	70.88	52.94	70.59	82.86	80.16	60.00	80.00	100.00	58.23	28.57	57.14	85.71
**LDA**	65.09	47.06	64.71	80.00	75.56	45.00	75.00	100.00	50.80	14.29	50.00	85.71
	Monte-Carlo repeated double cross validation (%)											
	Accuracy				Sensitivity				Specificity			
Model	Mean	Min	Median	Max	Mean	Min	Median	Max	Mean	Min	Median	Max
**Random Forest**	50.52	26.47	50.00	73.53	64.09	35.71	64.29	94.74	36.72	5.88	37.50	64.29
**LDA**	49.89	32.35	50.00	72.73	64.70	38.46	65.00	90.00	34.70	0.00	33.33	81.82

**Fig 2:**
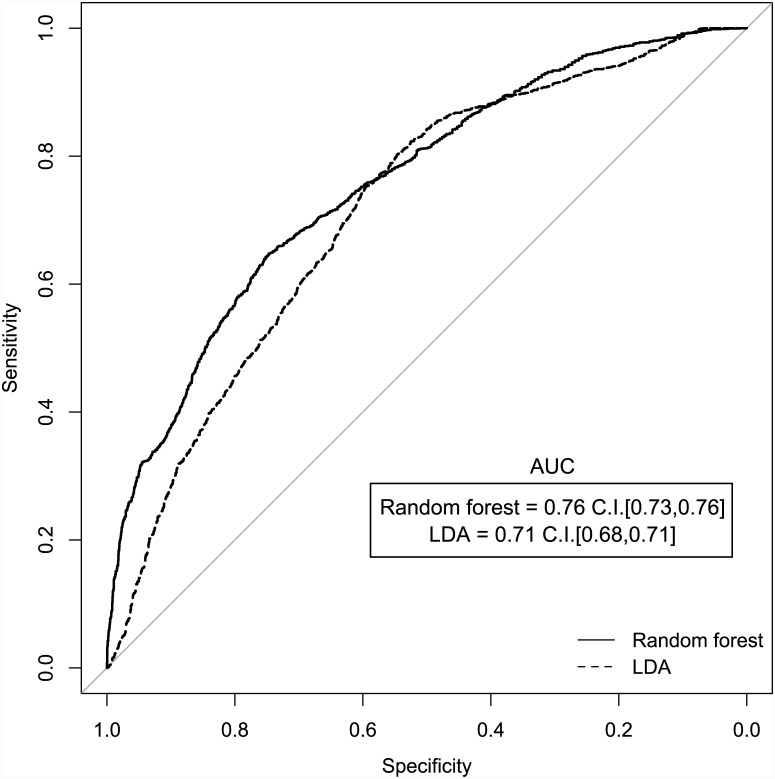
Receiver operating characteristic curve (ROC) for the random forest (RF) and linear discriminant analysis (LDA) models built using repeated double cross-validation to classify patients with prostate cancer and cancer-free controls based on PSA levels and VOCs in urine headspace.

Using repeated 10-fold cross-validation, patients could be classified into prostate cancer and cancer-free groups with mean accuracies of 74% and 65%, using Random Forest and Linear Discriminant Analysis, respectively. With a Monte Carlo random permutation of class labels, the mean accuracies fell to 56% and 48%, using Random Forest and Linear Discriminant Analysis, respectively. Hence the results obtained by the two cross-validation schemes were superior to those that could be expected by chance. Using repeated double cross-validation, patients could be classified with mean accuracies of 71% and 65%, using Random Forest and Linear Discriminant Analysis classification techniques, respectively. Fig 2 presents the receiver operating characteristic curve for the double repeated cross-validation analysis. The Monte Carlo random permutation of class labels reduced the diagnostic performance of the models to give mean test accuracies of 51 and 50% by random forest and linear discriminant analysis, respectively. These latter Monte Carlo accuracies closely align with the expected classification accuracy of 52% under chance agreement with fixed marginal frequencies and indicate that the classification rate in the authentic models (71% and 65%) is not an artefact of the modelling process.

## Discussion

For the first time, the VOC profiles of urinary headspace were studied from men with a suspicion of prostate cancer investigated by TRUS-guided needle prostate biopsy to confirm, or rule out, a diagnosis of prostate cancer. The objective of this reported work was to assess if the VOC profiles from urine headspace can help supplement current clinical tests to improve the stratification of men at risk of prostate cancer and, thereby, help to reduce the number of patients subjected to unnecessary needle biopsy.

There have been limited mass spectrometry studies published on volatile analyses of urine for prostate cancer detection. In a pilot study by our group we assessed the VOC profiles of urine from 24 asymptomatic men and 13 men with prostate cancer [48]. 21 VOCs were found to be positively associated with urine from prostate cancer patients. Similarity coefficients were calculated for each of the samples based on the presence or absence of these compounds in both groups. When applied to a multivariate discriminant analysis rule, these similarity coefficients allowed for discrimination of cases with 92.3% sensitivity and 96.3% specificity. Although promising, that study was too small to draw any robust conclusions and the use of asymptomatic men as controls meant that it did little to address one of the present clinical challenges of distinguishing patients with prostate cancer from those with non-malignant urological problems that often result in a raised PSA level; in the earlier study only one of the controls have a raised PSA.

Peng *et al*., 2010 tested the exhaled VOC profiles of healthy controls (n = 22) and patients with lung (n = 30), breast (n = 22), colorectal (n = 26), and prostate cancer (n = 18) [49]. The authors found that toluene, 2-amino-5-isopropyl-8-methyl-1-azulenecarbonitrile, p-xylene, and 2,2-dimethyl-decane showed no overlap in abundance between the healthy controls and patients with prostate cancer. However, they excluded compounds present in <80% of both cancer and control groups from the analysis. This is likely to have resulted in the loss of compounds with better discriminating power than those reported above which gave rise to close clusters in compound abundances between the prostate cancer and control groups. In addition, Peng *et al*. used a relatively small study size and compared cancerous groups to healthy cases, which are not suitable controls. A study published in Nature generated significant interest in the molecule sarcosine as a potential biomarker of aggressive prostate cancers [23]. Significantly higher levels of sarcosine were reported in both urine sediments and supernatants from biopsy-positive prostate cancer patients (*n* = 44) compared to biopsy-negative controls (*n* = 51). However, the predictive value of sarcosine was modest with an overall area under the curve (AUC) of the receiver operating characteristic (ROC) curve of 0.71 for urine sediments and 0.67 for supernatants. Further studies looking to validate this work proved disappointing [24–26]. On identifying the limited capabilities of sarcosine, Wu and co-workers went on to investigate other metabolomic markers in urine from 20 patients with prostate cancer, 8 patients with benign prostatic hypertrophy, and 20 healthy men [26]. They used microwave-assisted derivatization prior to GC/MS analyses for the detection of higher molecular weight compounds such as amino acids, organic acids, carbohydrates and fatty acids. Higher levels of the organic acids: dihydroxybutanoic acid and xylonic acid and lower levels of pyrimidine and the carbohydrates: xylopyranose and ribofuranoside were observed in the prostate cancer group. A diagnostic model, based on these 5 marker metabolites, reported an AUC value of 0.825 of the ROC curve. Again the main limitation of this work is the lack of adequate and suitable controls. Much further work is required in large, multi-centre studies by independent research groups if robust biomarkers for prostate cancer are ever to be found considering the past failures to corroborate initially “promising” biomarkers, with PSA and sarcosine being prime examples. In reality the majority of novel biomarkers reported in the literature fail the next hurdle to validate their potential for the diagnosis or management of cancer. Prensner *et al*., listed five common reasons for this: the lack of a robust test protocol for reproducibility, a biased comparison of groups in the study (case versus controls), undefined or inappropriate clinical role of the biomarker, a statistically underpowered study size, and inappropriate statistical analysis, including over fitting of the data [50]. Until results can be validated in separate trials, suitable cross-validation of the statistical analysis should be applied as a precautionary approach to estimate the predictive accuracy (and hence validity) of the biomarker(s) on new cases drawn from the same patient groups. Rosenberg *et al*., have introduced the application of a double cross-validation scheme on proteomics data from human prostate and colon tumours [51]. In this current study, the classification models were validated using repeated and repeated double cross-validation. Nevertheless, the results reported here should still be treated with caution given this is a small study that could be unduly affected by random or non-random permutations and confounding factors. The VOC model was based on the presence or absence of four volatile compounds: 2,6-dimethyl-7-octen-2-ol, pentanal, 3-octanone, and 2-octanone. Except for pentanal, all of these compounds were down-regulated and/or less frequently present in the urine samples from prostate cancer patients. The production of aldehydes has been linked with cancer and inflammatory conditions via the excessive production of reactive oxygen species (ROS) known to induce lipid peroxidation [52]. This may explain the higher incidence of pentanal detected in the urine samples of patients with prostate cancer. In agreement with our findings, other metabolomic studies have also commonly observed a trend of decreased production (down-regulation) of certain metabolites in cancer groups compared to control groups [53, 54]. A possible explanation for this trend may be that cancerous cells are utilising some of these metabolites to meet demands for increased energy consumption and converting these compounds to other substances that could not be detected by GC/MS. S2 Table lists all the compounds found which adds to our knowledge of compounds found in basified urine from urological controls and cancer patients. This will be useful for comparisons in other studies measuring volatiles from urine.

The results we report here on the discriminating capabilities of urine VOCs are somewhat inconclusive, but they mirror the findings of other groups that have tested potential biomarkers in urine and blood (PCA3 [55], multiplex urine RNA based biomarkers [56], and the Prostate Health Index based on PSA and its derivative [–2]proPSA and %fPSA [57, 58]) for the discrimination of prostate cancer patients from controls. It is very important that the study cohorts accurately reflect the specific patient population for which the biomarker test is intended. Therefore, we recruited controls from the urology clinic on the day of their prostate biopsy, who were being followed up for symptoms suspicious of prostate cancer. Indeed, all patients in this study had either elevated PSA levels or abnormal findings on a digital rectal examination. Based on PSA levels alone, patients could be classified with mean accuracies of 61% and 63% using RF and LDA classification techniques, respectively, with repeated double cross-validation. It is clinically challenging to differentiate these non-cancerous patients with urological symptoms from those with prostate cancer. It was also challenging to discriminate between these two groups based on urinary VOCs as this gave similar classification results to that obtained with PSA levels. Based on the presence or absence of four compounds, urine sample were classified with mean accuracies of 65% and 63%, using RF and LDA classification techniques, respectively, with repeated double cross-validation. Combining PSA levels with urinary VOCs, only gave a marginal improvement in the classification of patients, reporting mean accuracies of 71% and 65% using RF and LDA classification techniques, respectively, with repeated double cross-validation.

These two techniques were chosen because of their complementary nature i.e. LDA is a single classifier which uses a linear decision boundary and has the virtue of simplicity when it works, whereas, and in contrast, the Random Forest is a powerful ensemble approach which may do well when complex interactions may be needed to obtain good predictive accuracy. In this current study, the classification models were validated using repeated and repeated double cross-validation. Repeated double cross-validation (rdCV) is a systematic procedure which repeatedly randomly splits the data into a calibration sample for model development and into a holdout sample for model evaluation, and provides a realistic estimation of model accuracy when applied to new observations drawn from the same homogenous population. Nevertheless, the results reported here should still be treated with caution given this is a small study which may not adequately capture the diversity in the population and which may be subject to confounding factors.

A multiplatform method that combines volatile analyses with analyses of non-volatile compounds (using nuclear magnetic resonance spectroscopy or high-performance liquid chromatography/mass spectrometry based approaches) will help achieve a more comprehensive understanding of the metabolic characteristics of prostate cancer and may help clarify the metabolic pathways associated with aggressive forms of cancer. The current data indicate that VOC analyses may be used in addition to PSA testing in finding patients with prostate cancer. Future work should consider methods to optimise the results and explore other means of extracting VOCs from urine samples such as derivatization.

A limitation of this study was that urine samples were obtained at different times of the day, therefore varying in concentration. The collection of first pass urine would have minimised urine dilution and differences in urine concentration between study participants, however this would have also hindered the collection of samples. Future studies of this kind should make effort to measure the levels of urinary creatinine or urine osmolality to determine the degree of urine dilution. This can help define an acceptable range of urine concentrations for the analysis of headspace VOCs or provide a means to correct for urine dilution.

It is also important to note that the biopsy result cannot exclude the presence of prostate cancer completely in these patients, but can only confirm that there was no cancer found in the tissue samples taken. Therefore a possibility still remains that some patients were incorrectly categorized as negative for prostate cancer and this could have impacted negatively on the diagnostic capability of the models.

Biomarker research normally focuses on early disease diagnosis but it has been argued that, for prostate cancer, the greatest unmet clinical need is to distinguish low-risk or slow-growing cancers from the aggressive ones [50]. The identification and validation of prognostic and predictive biomarkers will help reduce unnecessary interventions that may cause more harm than good, monitor progression during “watchful waiting” and target treatment for those patients who are most likely to benefit [50]. There needs to be more work undertaken to lead to an improved method for identifying aggressive tumours. A much larger study is warranted to investigate this.

## Conclusion

Urology patients with elevated PSA levels would normally undergo a TRUS-guided needle prostate biopsy to confirm or exclude a diagnosis of prostate cancer. In the population we studied, the classification of patients with urine VOC testing was comparable to PSA level testing. Combining PSA levels with urinary VOCs resulted in a marginal improvement in test performance. These results are encouraging and suggest that there are other metabolomic avenues worth exploring which could help improve the stratification of men at risk of prostate cancer requiring follow-up. This study also adds to our knowledge of compounds found in alkaline urine, from controls and cancer patients, which will be useful for comparisons in other studies investigating volatiles from urine.

## Supporting Information

S1 Table Summary of urological problems and co-morbidities and medication of study participants. (DOCX)

S2 Table Volatile organic compounds in urine library, tentatively identified by relative ion abundances detected (mass spectra). (DOCX)

S3 Table Data source. (CSV)
