# Supplementary material for: Urinary Volatile Organic Compounds for the Detection of Prostate Cancer
Source: PLoS One. 2015 Nov 24;10(11):e0143283. doi: 10.1371/journal.pone.0143283 (PMC4657998; doi:10.1371/journal.pone.0143283)
Supplement: S1 Table — (DOCX) [file pone.0143283.s001.docx]

Supplementary Table 1 Summary of urological problems and co-morbidities and medication of study participants

|  | **Patient group, cases (%)** | | | |
| --- | --- | --- | --- | --- |
|  | Non-cancerous controls  (n= 43) | | Prostate cancers  (n= 59) | |
| **Indication/ reason for referral** |  | |  | |
| Bladder outflow symptoms | 9 | 21 | 22 | 37 |
| Prostatomegaly | 15 | 35 | 21 | 36 |
| Raised PSA | 39 | 91 | 56 | 95 |
| Haematuria | 7 | 16 | 5 | 8 |
| Previous TRUS/TRUP | 3 | 7 | 4 | 7 |
| Known prostate cancer | 0 | 0 | 2 | 3 |
| **Co-morbidities** |  |  |  |  |
| Hypertension | 13 | 30 | 20 | 34 |
| Ischaemic heart disease | 3 | 7 | 11 | 19 |
| Cerebrovascular disease | 2 | 5 | 3 | 5 |
| Asthma/COPD | 1 | 2 | 5 | 8 |
| Diabetes (Type 1/2) | 4/0 | 9.4/0 | 2/1 | 3/2 |
| Rheumatological disease (RA/OA) | 0 | 0 | 1/4 | 1/7 |
| Parkinson's disease | 0 | 0 | 2 | 3 |
| Depression | 0 | 0 | 1 | 2 |
| Epilepsy | 0 | 0 | 1 | 2 |
| Inflammatory bowel disease | 0 | 0 | 2 | 3 |
| **Medication (any therapy within class)** |  |  |  |  |
| For poor outflow | 13 | 30 | 9 | 15 |
| Antihypertensive | 13 | 30 | 24 | 41 |
| Anti-anginals | 5 | 12 | 5 | 8 |
| Diuretics | 2 | 5 | 5 | 8 |
| Statins | 11 | 26 | 16 | 27 |
| Proton pump inhibitors | 5 | 12 | 9 | 15 |
| Bronchodilators | 2 | 5 | 5 | 8 |
| Aspirin | 8 | 19 | 14 | 24 |
| Clopidogrel | 0 | 0 | 2 | 3 |
| Oral hyoglycaemics | 4 | 9 | 1 | 2 |
| Insulin | 0 | 0 | 2 | 3 |
| NSAIDs | 4 | 9 | 2 | 3 |
| Anti-Parkinsonism | 0 | 0 | 1 | 2 |
| Antidepressants | 0 | 0 | 2 | 3 |
| Anticonvulsants | 0 | 0 | 1 | 2 |
| Mesalazine | 0 | 0 | 2 | 3 |
| Warfarin | 0 | 0 | 3 | 5 |
| Prednisolone | 0 | 0 | 1 | 2 |
