# Supplementary material for: Urinary Volatile Organic Compounds for the Detection of Prostate Cancer
Source: PLoS One. 2015 Nov 24;10(11):e0143283. doi: 10.1371/journal.pone.0143283 (PMC4657998; doi:10.1371/journal.pone.0143283)
Supplement: S2 Table — (DOCX) [file pone.0143283.s002.docx]

Supplementary Table 2 Volatile organic compounds in urine library, tentatively identified by relative ion abundances detected (mass spectra)

| Retention Time (min) | Compound | Most prominent ions | | | | Ion ratios | | |
| --- | --- | --- | --- | --- | --- | --- | --- | --- |
|  |  | M1 | M2 | M3 | M4 | R2 | R3 | R4 |
| 5.59 | trimethylamine | 58 | 42 | 59 | 30 | 0.31 | 0.26 | 0.15 |
| 5.66 | methanethiol | 47 | 48 | 45 | 18 | 0.82 | 0.54 | 0.25 |
| 6.61 | ethanol | 31 | 45 | 46 | 29 | 0.65 | 0.32 | 0.20 |
| 6.92 | furan | 39 | 68 | 38 | 40 | 0.78 | 0.20 | 0.14 |
| 7.11 | 2-propenal | 27 | 56 | 55 | 26 | 1.00 | 0.65 | 0.61 |
| 7.21 | propanal | 29 | 58 | 28 | 27 | 0.95 | 0.66 | 0.44 |
| 7.41 | acetone | 43 | 58 | 42 | 44 | 0.23 | 0.05 | 0.05 |
| 7.58 | isopropanol | 45 | 43 | 41 | 39 | 0.13 | 0.10 | 0.07 |
| 7.80 | carbon disulfide | 76 | 44 | 78 | 38 | 0.09 | 0.09 | 0.03 |
| 8.19 | dichloromethane | 84 | 49 | 86 | 51 | 0.96 | 0.66 | 0.30 |
| 8.39 | acetic anhydride | 43 | 42 | 29 | 28 | 0.25 | 0.16 | 0.06 |
| 9.01 | 2-methylpropanal | 43 | 41 | 72 | 27 | 0.79 | 0.53 | 0.26 |
| 9.07 | hexane | 57 | 41 | 56 | 43 | 0.69 | 0.57 | 0.50 |
| 9.57 | 1-propanol | 31 | 59 | 42 | 29 | 0.19 | 0.18 | 0.13 |
| 10.03 | allyl mercaptan | 74 | 39 | 41 | 45 | 0.74 | 0.72 | 0.27 |
| 10.28 | 2,3-butanedione | 43 | 86 | 42 | 15 | 0.23 | 0.08 | 0.03 |
| 10.32 | 3-methylfuran | 82 | 53 | 81 | 39 | 0.68 | 0.57 | 0.28 |
| 10.48 | 1-propanethiol | 43 | 41 | 76 | 42 | 0.99 | 0.89 | 0.70 |
| 10.53 | ethyl acetate | 43 | 45 | 29 | 61 | 0.14 | 0.12 | 0.12 |
| 10.56 | 2-butanone | 43 | 72 | 29 | 57 | 0.30 | 0.14 | 0.09 |
| 11.61 | formic acid | 46 | 45 | 29 | 44 | 0.77 | 0.50 | 0.20 |
| 12.00 | 2-methyl-1-propanol | 43 | 41 | 42 | 33 | 0.82 | 0.62 | 0.40 |
| 12.37 | benzene | 78 | 77 | 52 | 51 | 0.36 | 0.31 | 0.28 |
| 12.51 | acetic acid | 43 | 45 | 60 | 42 | 0.85 | 0.61 | 0.17 |
| 12.54 | 3-methylbutanal | 44 | 41 | 43 | 58 | 0.89 | 0.69 | 0.42 |
| 12.81 | 2-methylbutanal | 57 | 41 | 58 | 29 | 0.85 | 0.61 | 0.40 |
| 13.04 | 3-methyl-2-butanone | 43 | 41 | 86 | 39 | 0.24 | 0.18 | 0.12 |
| 13.28 | 3-methyl-3-buten-2-one | 41 | 43 | 84 | 69 | 0.91 | 0.76 | 0.60 |
| 13.44 | 1-butanol | 56 | 41 | 43 | 31 | 0.59 | 0.53 | 0.42 |
| 13.50 | 2-ethylfuran | 81 | 96 | 53 | 39 | 0.36 | 0.25 | 0.13 |
| 13.54 | allyl methyl sulfide | 88 | 73 | 45 | 46 | 0.90 | 0.72 | 0.36 |
| 13.83 | 2,5-dimethylfuran | 96 | 95 | 43 | 53 | 0.89 | 0.59 | 0.57 |
| 13.98 | 2-pentanone | 43 | 86 | 41 | 71 | 0.22 | 0.12 | 0.10 |
| 14.16 | 2,3-pentanedione | 43 | 57 | 29 | 100 | 0.65 | 0.42 | 0.37 |
| 14.20 | pentanal | 44 | 29 | 58 | 41 | 0.41 | 0.40 | 0.37 |
| 14.87 | acetic acid, methyl ester | 43 | 31 | 74 | 42 | 0.12 | 0.09 | 0.09 |
| 15.94 | dimethyl disulfide | 94 | 45 | 79 | 46 | 0.52 | 0.51 | 0.27 |
| 16.08 | 3-methyl-1-butanol | 55 | 70 | 42 | 41 | 0.81 | 0.63 | 0.58 |
| 16.19 | 2-methyl-1-butanol | 57 | 56 | 41 | 70 | 0.93 | 0.84 | 0.44 |
| 16.42 | pyridine | 79 | 52 | 51 | 50 | 0.53 | 0.26 | 0.20 |
| 16.45 | 3-penten-2-one | 69 | 41 | 84 | 43 | 0.74 | 0.44 | 0.43 |
| 16.58 | toluene | 91 | 92 | 65 | 39 | 0.62 | 0.11 | 0.07 |
| 16.58 | 2-methyl-2-butenal | 84 | 55 | 39 | 41 | 0.84 | 0.39 | 0.38 |
| 16.73 | 3-methyl-2-pentanone | 43 | 57 | 41 | 72 | 0.37 | 0.32 | 0.22 |
| 16.75 | 2-methyl-2-butenenitrile | 54 | 81 | 41 | 53 | 0.78 | 0.68 | 0.48 |
| 17.11 | 2-methylfuran | 82 | 53 | 81 | 39 | 0.67 | 0.55 | 0.30 |
| 17.17 | 1-methyl-1H-pyrrole | 81 | 80 | 39 | 53 | 0.67 | 0.36 | 0.30 |
| 17.20 | 3-methyl-3-buten-1-ol | 41 | 56 | 39 | 68 | 0.77 | 0.70 | 0.67 |
| 17.28 | piperidine | 84 | 85 | 56 | 44 | 0.51 | 0.45 | 0.31 |
| 17.45 | 1-pentanol | 42 | 55 | 41 | 70 | 0.88 | 0.64 | 0.63 |
| 17.64 | 1-methyl-piperidine | 98 | 43 | 99 | 42 | 0.41 | 0.37 | 0.31 |
| 17.85 | 3-hexanone | 43 | 57 | 71 | 100 | 0.77 | 0.61 | 0.50 |
| 17.90 | 3-methylene-2-pentanone | 55 | 43 | 83 | 98 | 0.80 | 0.56 | 0.25 |
| 18.02 | propanethioc acid, S-methyl ester | 57 | 104 | 29 | 43 | 0.69 | 0.63 | 0.44 |
| 18.03 | 2-hexanone | 43 | 58 | 100 | 85 | 0.61 | 0.23 | 0.15 |
| 18.04 | 2,4-dimethyl-3-pentanone | 43 | 71 | 41 | 114 | 0.34 | 0.21 | 0.12 |
| 18.10 | methyl isobutyl ketone | 43 | 58 | 57 | 100 | 0.62 | 0.46 | 0.24 |
| 18.36 | 2,4-pentadienenitrile | 52 | 79 | 51 | 50 | 0.42 | 0.34 | 0.17 |
| 18.43 | hexanal | 44 | 41 | 56 | 43 | 0.95 | 0.90 | 0.83 |
| 19.06 | pyrrole | 67 | 41 | 39 | 40 | 0.51 | 0.49 | 0.44 |
| 19.20 | methallyl cyanide | 41 | 81 | 39 | 54 | 0.69 | 0.43 | 0.41 |
| 19.62 | butanoic acid | 60 | 73 | 45 | 42 | 0.33 | 0.23 | 0.21 |
| 19.75 | 5-methyl-3-hexanone | 57 | 85 | 41 | 29 | 0.30 | 0.29 | 0.21 |
| 19.80 | 4-methyl-3-hexanone | 57 | 29 | 41 | 114 | 0.23 | 0.12 | 0.10 |
| 20.07 | 1-ethyl-1H-pyrrole | 80 | 95 | 67 | 53 | 0.85 | 0.24 | 0.22 |
| 20.19 | methyl-pyrazine | 94 | 67 | 39 | 40 | 0.41 | 0.18 | 0.16 |
| 20.34 | ethylbenzene | 91 | 106 | 77 | 51 | 0.36 | 0.11 | 0.10 |
| 20.37 | 3-methyl-2-hexanone | 43 | 72 | 71 | 41 | 0.47 | 0.14 | 0.14 |
| 20.80 | N,N-dimethylformamide | 73 | 44 | 42 | 28 | 0.88 | 0.41 | 0.11 |
| 20.82 | 3-furaldehyde | 96 | 95 | 39 | 38 | 0.98 | 0.36 | 0.11 |
| 20.95 | 2-methyl-N-(2-methylpropyl)-1-propanamine | 86 | 57 | 30 | 41 | 0.38 | 0.28 | 0.25 |
| 21.19 | 4-heptanone | 71 | 43 | 114 | 41 | 0.97 | 0.30 | 0.26 |
| 21.36 | N-methylformamide | 59 | 30 | 28 | 29 | 0.38 | 0.29 | 0.13 |
| 21.47 | xylene* | 91 | 106 | 105 | 77 | 0.54 | 0.22 | 0.12 |
| 21.49 | 1-hexanol | 56 | 55 | 43 | 42 | 0.50 | 0.45 | 0.36 |
| 21.73 | 2-methylbutanoic acid | 74 | 57 | 41 | 87 | 0.53 | 0.49 | 0.26 |
| 21.77 | 3-heptanone | 57 | 29 | 85 | 41 | 0.40 | 0.35 | 0.29 |
| 21.78 | 2-n-butylacrolein | 41 | 97 | 39 | 43 | 0.82 | 0.65 | 0.65 |
| 21.83 | styrene | 104 | 103 | 78 | 51 | 0.46 | 0.41 | 0.20 |
| 22.04 | allyl isothiocyanate | 99 | 41 | 39 | 72 | 0.62 | 0.58 | 0.40 |
| 22.10 | phellandrene* | 93 | 91 | 77 | 92 | 0.60 | 0.37 | 0.35 |
| 22.11 | 2-heptanone | 43 | 58 | 71 | 41 | 0.65 | 0.16 | 0.13 |
| 22.33 | heptanal | 70 | 44 | 41 | 43 | 0.98 | 0.85 | 0.72 |
| 22.36 | 3-methyl-pyridine | 93 | 66 | 92 | 65 | 0.40 | 0.38 | 0.34 |
| 22.47 | 2,4-dimethylthiophene | 111 | 112 | 97 | 45 | 0.85 | 0.64 | 0.18 |
| 22.55 | 3-methyl-1H-pyrrole | 80 | 81 | 53 | 51 | 0.64 | 0.29 | 0.10 |
| 22.59 | xylene* | 91 | 106 | 105 | 92 | 0.48 | 0.26 | 0.11 |
| 22.62 | 3,7-dimethyl-1,6-octadiene | 41 | 55 | 67 | 69 | 0.82 | 0.70 | 0.65 |
| 22.67 | 2,5-dimethylpyrazine | 108 | 42 | 39 | 40 | 0.70 | 0.24 | 0.17 |
| 23.01 | pentanoic acid | 60 | 73 | 41 | 43 | 0.43 | 0.33 | 0.25 |
| 23.11 | cyclohexanone | 55 | 42 | 98 | 69 | 0.68 | 0.59 | 0.45 |
| 23.29 | 3,7-dimethyl-2-octene | 70 | 41 | 55 | 69 | 0.78 | 0.70 | 0.60 |
| 23.37 | 2-acetyl-1-pyrroline | 43 | 41 | 83 | 42 | 0.53 | 0.46 | 0.26 |
| 23.47 | methyl propyl disulfide | 122 | 80 | 43 | 41 | 0.87 | 0.53 | 0.51 |
| 23.57 | methional | 48 | 104 | 76 | 47 | 0.83 | 0.47 | 0.45 |
| 23.66 | 2,2-dimethyl-butanoic acid | 71 | 43 | 41 | 88 | 0.92 | 0.31 | 0.23 |
| 23.74 | 2-acetylfuran | 95 | 110 | 39 | 38 | 0.42 | 0.21 | 0.07 |
| 23.81 | 2-methyl-4-heptanone | 57 | 43 | 41 | 85 | 0.76 | 0.60 | 0.59 |
| 23.90 | 1-(3-ethylcyclobutyl)-ethanone | 43 | 71 | 97 | 55 | 0.75 | 0.69 | 0.53 |
| 24.02 | 3-methyl-2-heptanone | 43 | 72 | 41 | 85 | 0.59 | 0.18 | 0.17 |
| 24.15 | 3-hepten-2-one | 55 | 97 | 43 | 112 | 0.68 | 0.49 | 0.36 |
| 24.19 | phellandrene* | 93 | 91 | 77 | 79 | 0.44 | 0.42 | 0.31 |
| 24.26 | decane | 57 | 43 | 41 | 71 | 0.92 | 0.46 | 0.30 |
| 24.36 | 4-methyl-1-(1-methylethyl)-cyclohexene | 95 | 81 | 67 | 138 | 0.70 | 0.34 | 0.32 |
| 24.68 | pinene* | 93 | 41 | 69 | 91 | 0.39 | 0.33 | 0.28 |
| 24.78 | 2-pentylfuran | 81 | 82 | 138 | 53 | 0.25 | 0.22 | 0.13 |
| 24.89 | 2-heptanal | 41 | 55 | 39 | 83 | 0.74 | 0.62 | 0.61 |
| 24.96 | butanoic acid, butyl ester | 71 | 89 | 56 | 43 | 0.63 | 0.48 | 0.43 |
| 24.97 | 2-methyl-5-(methylthio)-furan | 128 | 113 | 85 | 45 | 0.86 | 0.72 | 0.15 |
| 25.08 | 6-methyl-3-heptanone | 43 | 57 | 72 | 81 | 0.83 | 0.74 | 0.57 |
| 25.12 | methoxy-phenyl-oxime | 133 | 151 | 135 | 134 | 0.68 | 0.23 | 0.14 |
| 25.13 | 1,5,5-trimethyl-3-methylene-cyclohexene | 121 | 93 | 136 | 79 | 0.65 | 0.44 | 0.42 |
| 25.25 | phellandrene* | 93 | 91 | 41 | 77 | 0.57 | 0.41 | 0.40 |
| 25.34 | dimethyl trisulfide | 126 | 79 | 45 | 47 | 0.44 | 0.38 | 0.27 |
| 25.43 | 3-ethylcyclopentanone | 83 | 55 | 41 | 112 | 0.64 | 0.49 | 0.48 |
| 25.47 | 3-octanone | 57 | 43 | 72 | 99 | 1.00 | 0.59 | 0.49 |
| 25.60 | 6-methyl-5-hepten-2-one | 43 | 108 | 41 | 69 | 0.59 | 0.55 | 0.41 |
| 25.69 | 1-methyl-4-(1-methylethyl)-1,3-cyclohexadiene | 121 | 93 | 91 | 136 | 0.87 | 0.50 | 0.46 |
| 25.73 | benzaldehyde | 106 | 77 | 105 | 51 | 1.00 | 0.79 | 0.41 |
| 25.86 | 5-methyl-2-furancarboxaldehyde | 109 | 110 | 53 | 51 | 0.91 | 0.54 | 0.16 |
| 25.91 | 1,4-cineole | 111 | 43 | 71 | 55 | 0.94 | 0.70 | 0.47 |
| 25.94 | trimethyl-pyrazine | 122 | 42 | 81 | 39 | 0.73 | 0.17 | 0.17 |
| 26.08 | octanal | 43 | 56 | 41 | 57 | 0.84 | 0.80 | 0.79 |
| 26.13 | 3-methyl-cyclohexanone | 69 | 112 | 41 | 42 | 0.47 | 0.42 | 0.32 |
| 26.23 | limonene* | 68 | 67 | 93 | 79 | 0.80 | 0.63 | 0.37 |
| 26.35 | cymene* | 119 | 134 | 91 | 117 | 0.28 | 0.25 | 0.15 |
| 26.39 | phellandrene* | 93 | 91 | 77 | 79 | 0.50 | 0.41 | 0.29 |
| 26.66 | dimethyl sulfone | 79 | 94 | 15 | 45 | 0.62 | 0.15 | 0.06 |
| 26.66 | 2-octanone | 43 | 58 | 41 | 59 | 0.73 | 0.24 | 0.16 |
| 26.76 | eucalyptol | 43 | 81 | 71 | 84 | 0.72 | 0.61 | 0.55 |
| 26.85 | 2-octanol | 45 | 55 | 43 | 41 | 0.26 | 0.20 | 0.19 |
| 27.05 | terpinene* | 93 | 91 | 136 | 77 | 0.55 | 0.41 | 0.37 |
| 27.14 | 2-ethyl-1-hexanol | 57 | 41 | 43 | 70 | 0.39 | 0.33 | 0.31 |
| 27.62 | 4-methyl-1-(1-methylethenyl)-cyclohexene | 79 | 93 | 107 | 136 | 0.59 | 0.54 | 0.48 |
| 27.68 | undecane | 57 | 43 | 41 | 71 | 0.98 | 0.56 | 0.46 |
| 27.69 | 2,2,6-trimethyl-cyclohexanone | 82 | 69 | 140 | 56 | 0.41 | 0.34 | 0.33 |
| 27.69 | 2-ethenyl-6-methyl-pyrazine | 120 | 52 | 18 | 119 | 0.51 | 0.49 | 0.34 |
| 27.91 | 4-methyl-3-pentenoic acid | 43 | 99 | 55 | 70 | 0.95 | 0.55 | 0.39 |
| 28.14 | phenol | 94 | 66 | 65 | 39 | 0.37 | 0.28 | 0.22 |
| 28.15 | 1-methyl-4-(1-methylethylidene)-cyclohexene | 121 | 93 | 136 | 91 | 0.99 | 0.77 | 0.53 |
| 28.37 | 1-octanol | 56 | 41 | 42 | 70 | 0.63 | 0.61 | 0.52 |
| 28.41 | 2,6-dimethyl-7-octen-2-ol | 59 | 55 | 43 | 41 | 0.25 | 0.24 | 0.22 |
| 28.55 | Isophorone* | 82 | 54 | 138 | 39 | 0.23 | 0.17 | 0.14 |
| 28.66 | a-methyl-a-(4-methyl-3-pentenyl)oxiranemethanol | 59 | 43 | 94 | 93 | 0.70 | 0.61 | 0.48 |
| 28.72 | 1-methyl-4-(1-methylethenyl)-benzene | 117 | 132 | 115 | 91 | 0.81 | 0.49 | 0.44 |
| 29.06 | 2-nonanone | 58 | 43 | 57 | 59 | 0.84 | 0.30 | 0.26 |
| 29.13 | 3,7-dimethyl-3-octanol | 73 | 69 | 55 | 43 | 0.52 | 0.32 | 0.30 |
| 29.24 | 3,7-dimethyl-1,6-octadien-3-ol | 71 | 93 | 55 | 41 | 0.75 | 0.69 | 0.61 |
| 29.31 | 4-nonanone | 43 | 71 | 99 | 58 | 0.97 | 0.55 | 0.46 |
| 29.35 | acetophenone | 105 | 77 | 120 | 51 | 0.95 | 0.44 | 0.43 |
| 29.57 | nonanal | 57 | 41 | 43 | 56 | 0.91 | 0.67 | 0.60 |
| 29.71 | 5-ethenyldihydro-5-methyl-2(3H)-furanone | 111 | 55 | 43 | 67 | 0.64 | 0.53 | 0.53 |
| 29.95 | diallyl disulphide | 41 | 39 | 45 | 81 | 0.50 | 0.21 | 0.20 |
| 30.17 | 2-methoxyphenol | 109 | 124 | 81 | 93 | 0.74 | 0.68 | 0.23 |
| 30.50 | 3,5-dimethyl-2-octanone | 72 | 43 | 57 | 41 | 0.78 | 0.28 | 0.21 |
| 30.61 | 1,7,7-trimethylbicyclo(2.2.1)hept-5-en-2-ol | 108 | 93 | 67 | 41 | 0.68 | 0.20 | 0.19 |
| 30.73 | 2-ethylhexanoic acid | 73 | 88 | 41 | 57 | 0.98 | 0.37 | 0.27 |
| 30.74 | 3-methyl-2-cyclohexen-1-one | 82 | 110 | 54 | 39 | 0.45 | 0.37 | 0.32 |
| 30.89 | 4-(methylthio)-butanenitrile | 61 | 115 | 41 | 62 | 0.54 | 0.40 | 0.27 |
| 30.91 | p-cresol | 107 | 108 | 77 | 79 | 0.77 | 0.28 | 0.21 |
| 31.01 | 1-methyl-4-(1-methylethyl)-3-cyclohexen-1-ol | 81 | 43 | 121 | 93 | 0.60 | 0.51 | 0.50 |
| 31.21 | 1,2,4,5-tetramethylbenzene | 119 | 134 | 91 | 120 | 0.44 | 0.16 | 0.09 |
| 31.39 | a,a,4-trimethyl-cyclohexanemethanol | 59 | 43 | 55 | 41 | 0.17 | 0.15 | 0.13 |
| 31.61 | 5-ethyldihydro-5-methyl-2(3H)-furanone | 99 | 43 | 56 | 55 | 0.56 | 0.19 | 0.19 |
| 31.87 | 1-piperidine acetonitrile | 123 | 124 | 41 | 83 | 0.44 | 0.36 | 0.33 |
| 31.87 | dodecane | 57 | 43 | 71 | 41 | 0.96 | 0.54 | 0.45 |
| 32.08 | 4-acetyl-1-methylcyclohexene | 43 | 95 | 138 | 67 | 0.92 | 0.70 | 0.61 |
| 32.29 | 2-nonanone | 58 | 43 | 71 | 59 | 0.62 | 0.42 | 0.29 |
| 32.42 | 5-methyl-2-(1-methylethyl)-cyclohexanol | 71 | 81 | 95 | 41 | 0.95 | 0.82 | 0.56 |
| 32.44 | 4,5,6,7-tetrahydro-3,6-dimethyl-benzofuran | 108 | 150 | 79 | 73 | 0.29 | 0.20 | 0.15 |
| 32.79 | Isophorone* | 82 | 138 | 39 | 54 | 0.31 | 0.20 | 0.11 |
| 33.01 | p-cymen-8-ol | 43 | 135 | 91 | 117 | 0.69 | 0.31 | 0.26 |
| 33.19 | azulene | 128 | 102 | 127 | 126 | 0.15 | 0.14 | 0.11 |
| 33.20 | methyl salicylate | 120 | 92 | 121 | 152 | 0.61 | 0.44 | 0.42 |
| 33.44 | pinocarvone | 53 | 81 | 108 | 41 | 0.71 | 0.60 | 0.51 |
| 33.66 | borneol | 95 | 110 | 41 | 67 | 0.22 | 0.19 | 0.12 |
| 33.90 | a-terpineol | 59 | 93 | 121 | 136 | 0.91 | 0.67 | 0.66 |
| 33.91 | 5-methyl-2-(1-methylethenyl)-cyclohexanone | 109 | 123 | 67 | 93 | 0.98 | 0.83 | 0.79 |
| 34.43 | 2,6,6-trimethyl-1,3-cyclohexadiene-1-carboxaldehyde | 107 | 91 | 121 | 105 | 0.89 | 0.54 | 0.49 |
| 34.67 | pulegone | 81 | 67 | 152 | 109 | 0.82 | 0.71 | 0.58 |
| 34.86 | 2,6,6-trimethyl-2,4-cycloheptadien-1-one | 107 | 150 | 108 | 91 | 0.61 | 0.54 | 0.41 |
| 34.87 | tridecane | 57 | 43 | 71 | 41 | 0.86 | 0.65 | 0.49 |
| 35.04 | 4-(1-methylethyl)-benzaldehyde | 133 | 105 | 148 | 77 | 0.80 | 0.79 | 0.35 |
| 35.64 | 1-decanol | 55 | 41 | 70 | 56 | 0.84 | 0.81 | 0.81 |
| 35.67 | 3-methyl-6-(1-methylethyl)-2-cyclohexen-1-one | 82 | 110 | 95 | 109 | 0.87 | 0.43 | 0.31 |
| 35.93 | carvone | 82 | 54 | 93 | 108 | 0.55 | 0.37 | 0.34 |
| 36.16 | 4-(1-methylethyl)-1-cyclohexene-1-carboxaldehyde | 109 | 79 | 81 | 41 | 0.54 | 0.54 | 0.47 |
| 36.58 | thymol | 135 | 150 | 91 | 115 | 0.29 | 0.19 | 0.16 |
| 36.71 | tetradecane | 57 | 43 | 71 | 41 | 0.73 | 0.51 | 0.35 |
| 37.15 | terpinene* | 93 | 121 | 136 | 68 | 0.83 | 0.64 | 0.45 |
| 38.66 | indole | 117 | 90 | 89 | 63 | 0.44 | 0.33 | 0.14 |
| 38.83 | damascenone | 69 | 121 | 105 | 41 | 0.80 | 0.29 | 0.28 |
| 39.11 | 3-(1-methyl-2-pyrrolidinyl)-pyridine | 84 | 133 | 162 | 42 | 0.48 | 0.19 | 0.17 |
| 39.26 | a-cedrene | 119 | 93 | 105 | 41 | 0.36 | 0.32 | 0.23 |
| 41.15 | 3-methyl-1H-indole | 130 | 131 | 77 | 64 | 0.43 | 0.18 | 0.17 |
| 41.17 | N-morpholinomethyl-isopropyl-sulfide | 100 | 43 | 41 | 56 | 0.11 | 0.08 | 0.08 |
| 41.42 | 2-nonanone | 58 | 43 | 59 | 57 | 0.39 | 0.26 | 0.23 |

*unknown isomer
